# Phytochemical Characterization and In Vitro Evaluation of the Anti-Sickle Cell Activity of Aqueous and Ethanolic Extracts of Two Medicinal Plants from Niger: *Flueggea virosa* (Roxb. ex Willd.) Royle and *Kigelia africana* (Lam.) Benth

**DOI:** 10.3390/plants12203522

**Published:** 2023-10-10

**Authors:** Harouna Dieté Souleymane, Alfa Keita Djibo, Sabo Haoua Seyni, Ousseini Zakaria, Andreea Veronica Botezatu, Rodica Mihaela Dinica, Adamou Ibrahim Maman Laouali, N’goran David Vincent Kouakou

**Affiliations:** 1 Laboratory of Natural Substances and Organic Synthesis, Department of Chemistry, Faculty of Sciences and Techniques, Abdou Moumouni University of Niamey, Niamey BP 10662, Niger; diete35261@gmail.com (H.D.S.); alfakeitad@yahoo.fr (A.K.D.); 2 Laboratory for Valorization of Agro-Resources, Biochemistry Section, Department of chemistry, Faculty of Sciences and Techniques, Abdou Moumouni University of Niamey, Niamey BP 10662, Niger; hseinisabo@yahoo.fr (S.H.S.); zousseini@yahoo.fr (O.Z.); 3 Department of Chemistry, Physics and Environment, Faculty of Sciences and Environment, “Dunarea de Jos” University of Galati, 800201 Galati, Romania; andreea.botezatu@ugal.ro; 4 Laboratory of Biostatistics, Department of Biology, Faculty of Sciences and Techniques, Abdou Moumouni University of Niamey, Niamey BP 10662, Niger; amamanlaouali@gmail.com; 5 Department of Training and Research in Agriculture and Animal Resources, National Polytechnic Institute of Félix Houphouët-Boigny, Yamoussoukro BP 1313, Côte d’Ivoire; david.kouakou@inphb.ci

**Keywords:** phytochemical screening, antisickling activity, *Flueggea virosa* (Roxb. ex Willd.) Royle, *Kigelia africana* (Lam.) Benth., Niger

## Abstract

Sickle cell anaemia is a hereditary blood disorder that attacks the red blood cells and deforms them, giving them a sickle shape. Sickle cell anaemia is a serious health problem in the West African country of Niger. Moreover, the cost associated with medical care is very high. The main objective of this study is to contribute to the valorisation of *Flueggea virosa* (Roxb. ex Willd.) Royle (aerial part), *Kigelia africana* (lam), and Benth (leaves) from Niger were used to treat sickle cell disease using aqueous and ethanolic extracts of phytochemical compounds. To achieve this objective, the evaluation of anti-sickle cell activity was carried out in vitro using the Emmel technique through the normalisation rate. The analyses showed that the aqueous and ethanolic extracts contained various classes of bioactive substances known for their valuable biological activities. The chemical composition rich in bioactive compounds led to very good results in biological assays. Thus, from a dose of 0.05 mg/mL, the ethanolic extracts of the two plants normalised up to 75% of the sickle cells. As the rate of normalisation was shown to be dose-dependent, at a dose of 10 mg/mL, the ethanolic extracts showed the best rates of sickle cell normalisation, with 95% for *F. virosa* and 93% for *K. africana*. Phytochemical screening was used to correlate the secondary metabolite and anti-sickle cell activities of the extracts from the two plants. These results may justify the use of these two species in traditional medicine for the treatment of sickle cell disease in Niger. The inclusion of these plants in phytomedicines could provide significant relief to people suffering from sickle cell disease.

## 1. Introduction

Sickle cell anaemia is a hereditary disease caused by a qualitative structural anomaly in haemoglobin. This effect is due to a point mutation in the sixth adenine codon and its replacement with thymine in the β-chain coding for globin. This DNA mutation is characterised by the substitution of an amino acid (glutamic acid) with valine at position 6 of the β chain. This anomaly is located on chromosome 11 [1,2]. According to the World Health Organization (WHO), it is the most common genetic disease in the world, affecting approximately 50 million people [3]. Every year, 300,000 children are born with a major haemoglobin abnormality, and there are more than 200,000 cases in Africa, with a prevalence of 13%. Haemoglobinopathies represent the most frequent category of clinically significant inherited diseases, with an enormous public health burden, and sickle cell disease is the most common haemoglobinopathy in sub-Saharan Africa [4]. In sub-Saharan Africa, where the majority of affected individuals are found, the disease is often fatal to children under the age of 5 [5]. In Niger, as in most Sahelian countries, sickle cell anaemia is one of the most impactful diseases. During the cold season (December to February), there are more sickle cell crises, with high infant morbidity and mortality in the most vulnerable groups, that is, mother–child pairs. Every year, 1000 new cases are recorded, with 0.5–1% of deaths occurring in 5% of the affected population. This condition affects 22% to 25% of the population of Niger [6]. Several therapeutic options, such as bone marrow transplants, repeated blood transfusions, and hydroxyurea, have been developed to combat sickle cell disease. These treatments are inaccessible to African populations because they are very expensive. They can also pose a risk of transmitting diseases such as hepatitis and HIV/AIDS [7,8,9]. In Niger, the population has acquired several years of experience with the traditional treatment of illnesses using plants, as reported by [10] in an ethnobotanical survey of traditional health practitioners in the Tillabery and Niamey regions.

Therefore, it is important to carry out scientific studies to prove and highlight the biological properties of these plants in terms of their anti-sickle cell activity due to their chemical composition. As the chemical composition has been described in other studies, this study was undertaken to evaluate the biological properties and propose the inclusion of these two medicinal plants in the Nigerien Pharmacopoeia.

## 2. Results

### 2.1. Extract Yield

The yields in (%) mass/mass of the aqueous and ethanolic crude extracts of both plants are given in Table 1. It can be seen that more substances were extracted in *F. virosa* using both solvents than in *K. africana* (Table 1).

### 2.2. Phytochemical Screening

Phytochemical screening of the crude aqueous and ethanolic extracts of *F. virosa* and *K. africana* revealed the richness of these plant species in chemical compounds. All nine chemical groups (alkaloids, sterols and triterpenes, saponosides, flavonoids, free quinones, tannins, anthocyanins, reducing sugars, and aromatic amino acids) were detected in *F. virosa* and *K. africana* (Table 2).

### 2.3. In Vitro Evaluation of the Antifalcemic Activity

**Figure 1:**
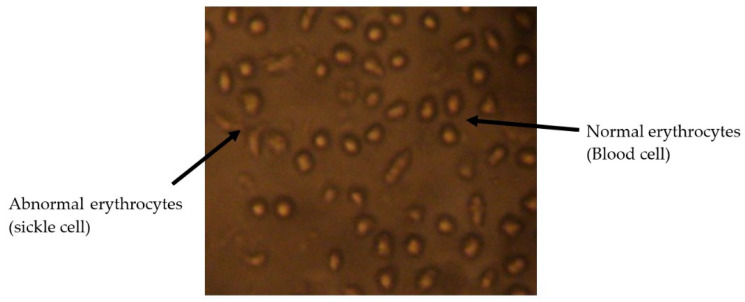
Morphology of sickle cells in untreated SS blood (400× magnification).

**Figure 2:**
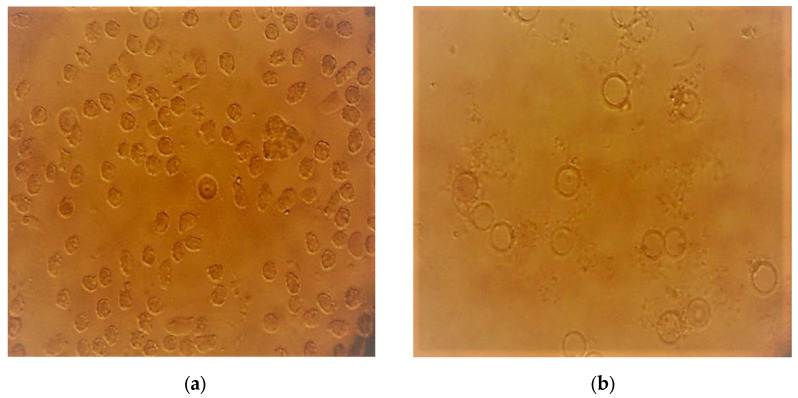
(**a**) Micrograph of SS blood sickle cells treated with ethanolic extract of *F. virosa* at a concentration of 2.5 mg/mL (400×), (**b**) Micrograph of SS blood sickle cells treated with ethanolic extract of *F. virosa* at a concentration of 2.5 mg/mL.

**Figure 3:**
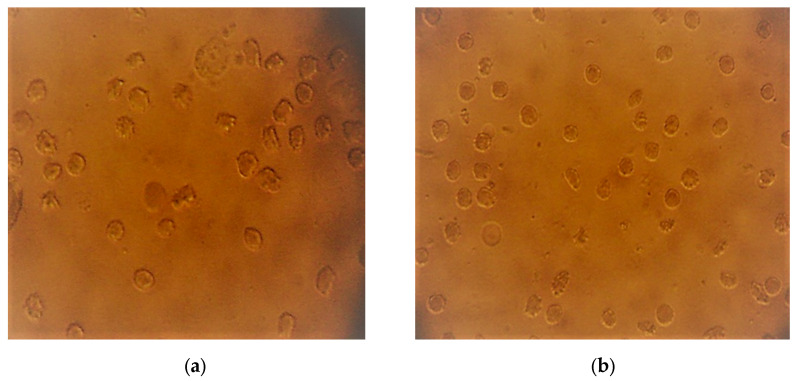
(**a**) Micrograph of sickle cells from SS blood treated with aqueous extract of *K. africana* at a concentration of 2.5 mg/mL (400×), (**b**) Micrograph of sickle cells from SS blood treated with ethanolic extract of *K. africana* at a concentration of 2.5 mg/mL (400×).

Figure 1, Figure 2 and Figure 3 present micrographs of sickle cells and erythrocytes from the control (SS blood not treated with the plant extract) and SS samples treated with aqueous and ethanolic extracts of *F. virosa* and *K. africana* at a concentration of 2.5 mg/mL. A concentration of 2.5 mg/mL was used to illustrate the shape of the red blood cells after treatment of the blood samples with the extracts (Figure 2 and Figure 3). Figure 1 shows that there were more sickle cells than normal erythrocytes in SS blood untreated with the crude extracts of the two plants; in contrast, in Figure 2 and Figure 3, there were more normal erythrocytes than sickle cells in SS samples treated with the aqueous and ethanolic extracts of *F. virosa* (Figure 2) and *K. africana* (Figure 3) at a concentration of 2.5 mg/mL.

**Figure 4:**
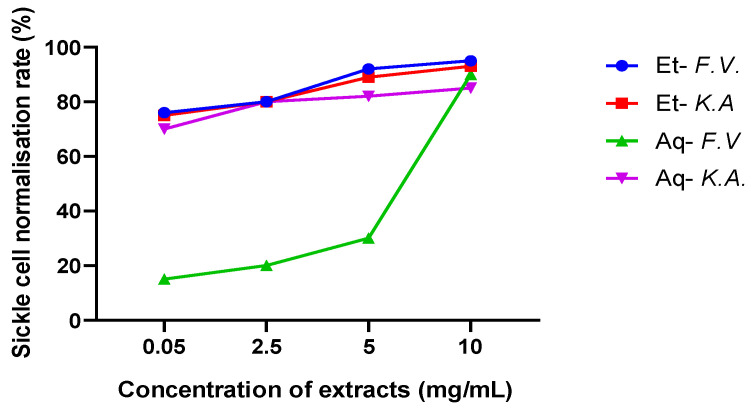
Normalisation rate of sickle cells in the presence of the extracts according to the concentrations of the crude extracts of *F. virosa* and *K. africana*. Legend: Et-*F.V.* ethanolic extract of *F. virosa*; ET-*K.A.* ethanolic extract of *K. Africana*; Aq-*F.V*. aqueous extract of *F. virosa;* Aq-*K.A*. aqueous extract of *K. africana*.

The high rates of normalisation of red blood cells in the SS blood samples treated with the extracts showed that the two plants contained substances that could return sickle cells to a normal form, under in vitro hypoxic conditions, compared with the negative control, for which the number of sickle cells was high. Generally, a plant is considered an anti-sickle cell when it can normalise 50% of the sickle cells. From the first dose (0.05 mg/mL), all ethanolic extracts of the two plants normalised the number of sickle cells by up to 75% (Figure 4). It should be noted that the greatest anti-sickle cell activity was observed more in the ethanolic extracts than in the aqueous extracts. The highest rates of sickle cell normalisation were obtained at a dose of 10 mg/mL, with a rate of 95% for the ethanolic extract of *F. virosa*, followed by that of the ethanolic extract of *K. africana* (93%) and then the normalisation rates for the aqueous extracts of *F. virosa* (90%) and *K. africana* (85%) (Table 3).

Although these two plants belong to different families, their normalization rates were statistically identical: *F. virosa* (62.25 ± 34.41%) and *K. africana* (81.75 ± 7.36%) were not significant, the variance was equal to 0.139 above the probability threshold (*p* > 0.05), and these two plants are in the same group (a) in terms of modalities.

In addition, depending on the solvent used for extraction, it was observed that the normalisation rate of ethanolic extracts (85 ± 8.1%) was statistically different from that of aqueous extracts (59 ± 31.68 %), and the variance equal to 0.041 was lower than the probability threshold (*p* < 0.05). These two extracts were obtained using different groups of modalities.

The difference observed between the rate of sickle cell normalisation in aqueous and ethanolic extracts of the two plants could be explained by the difference in the polarity of the solvents. These results indicate that the chemical groups responsible for the anti-sickle cell activity could be less polar compounds and more soluble in ethanol than in water.

## 3. Discussion

The average yield of the ethanolic extracts was higher than that of the aqueous extracts, indicating that extracted ethanol had more phytochemical compounds than water.

### 3.1. Phytochemical Screening

Phytochemical screening of *Flueggea virosa* by Benin [11] detected the presence of gallic and catechic tannins, flavonoids, and anthocyanins in *Flueggea virosa*. In this study, in addition to these compounds, alkaloids, quinone derivatives, triterpenes and sterols, saponosides, reducing compounds, and aromatic amino acids were identified in *F. virosa* from Niger (Table 4). This wealth of phytochemicals justifies the use of the leaves, twigs, and bark in Niger’s traditional medicine to treat illnesses, such as sickle cell anaemia, allergic and infectious dermatitis, stomach ache, and heart pain [10]. The plant is also used to treat fever, malaria, genital dysfunction, pain, diabetes, epilepsy, venereal disorders, diarrhoea, pneumonia, cough, AIDS, and as a contraceptive [12]. Various indolizidine alkaloids, including isomers and securin derivatives, have been isolated from the organs of *Flueggea virosa*, namely virosecurinine (0.5% in the leaves), viroallosecurinine, norsecurinine, and dihydronorsecurinine (virosine). Other alkaloids, such as hordenine and N-methyltetrahydro-β-carboline, have also been isolated. Other compounds isolated from *F. virosa* leaves include isocoumarin, bergenin, gallic acid, ellagic acid, and flavonoids (quercetin and rutin). The DPPH free radical scavenging assay of *F. virosa* showed that corilagin, rutin, and gallic acid displayed strong antioxidant activities, with DPPH radical scavenging capacities of 82.74%, 75.31% and 91.83% at a concentration of 200 μg/mL, respectively [13] (Table 4).

*F. virosa* twigs contain around 8% tannins [14,15]. Two dimeric indolizidine alkaloids, flueggines A and B, were isolated from the twigs and leaves of *Flueggea virosa*. Aqueous extracts of *F. virosa* root have demonstrated anti-inflammatory and antipyretic activities, with a significant effect observed at a dose of 400 mg/kg. Acute toxicity was assessed in rats, where the extract was administered orally at a dose of 10,000 mg/kg, and no deaths were recorded [16].

In the second plant, *Kigelia africana*, nine families of chemical compounds were identified during phytochemical screening (alkaloids, triterpenes and sterols, flavonoids, quinone derivatives, saponosides, tannins, anthocyanins, reducing compounds, and aromatic amino acids). The presence of alkaloids, sterols, triterpenes, and saponosides has already been reported in *K. africana* from Niger by [17]; however, these authors did not identify any tannins, flavonoids, quinones, or cyanogenic glycosides in the plant, compounds that were found in the samples investigated in the current study (Table 5). This could be due to differences in harvesting area or harvesting time. The richness of its compounds justifies its use in traditional Nigerien medicine, where the leaves, bark, and fruit are used in treating sickle cell disease, diabetes, wounds, dysentery, and gastritis [10]. Various chemical constituents, such as naphthaquinones, iridoids, fatty acids, norviburtinal, sterols, lignans, terpenoids, caffeic acid, flavonoids, and kigelinone, have been reported in *K. africana* [18,19]. In another study, the total phenolic content in the examined extract was found to be 3.53g of GA/100 g of the methanolic extract of *Kigelia africana* from India [20] (Table 5).

### 3.2. In Vitro Evaluation of the Antifalcemic Activity

The results in Figure 4 show that the percentages of normalisation of sickle cell (thus, the antifalcemic activity) were dose-dependent with the dose of the plant extract. Thus, the higher the concentration of the extract, the higher the normalization rate. Other authors have reported this fact in the literature [22,23]. Thus, [24] found anti-sickle cell activity in polar (aqueous, methanolic) and medium polar (ethyl acetate) crude extracts of *Ocimum canun* (Lamiaceae), with apolar (n-hexane and dichloromethane) extracts of the plant being without activity. According to these authors, anthocyanins and polar compounds in these extracts are responsible for the anti-sickle cell activities of the plant, with a maximum normalisation rate of 88.5% for sickle cells. Similarly, [25] found high anti-sickle cell activities with aqueous extracts of *Jatropha curcas* and *Dichrostachys cinerea,* with sickle cell inhibition rates of 97% and 91%, respectively, and an inhibition rate of only 32% for *Khaya senegalensis*. It was also found that the methanolic and ethyl acetate extracts were responsible for the antifalcemic activities of *Combretum glutinosom* leaves, with sickle cell conversion rates of 81% and 89%, respectively [26]. A high sickle cell normalisation rate (71%) was reported by [27] for the aqueous extract of a recipe (mixture of plants) used to combat sickle cell disease, in which *Cissus populnea* is the main plant. However, these authors found a higher normalisation rate (62%) for the chloroformic extract of *Cissus populnea* than for the aqueous extract (more polar) of the same plant (53%).

The antifalcemic activities of the polar extracts of medicinal plants have been attributed to certain molecules, such as polar, vitamin C and phenylalanine [28], vanillic acid [29], p-hydroxybenzoic acid [27,28,29,30], ortho-3,4-divalloylquinic acid, ortho-3,5 divalloylquinic acid, ortho-4,5-divalloylquinic acid [31], phenylalanine [30,32], and polar chemical compounds such as alkaloids [1], anthocyanins [1], flavonoids [26], quinones [28], tannins [33], organic acids [31], and aromatic amino acids [30,32]. The results of the phytochemical screening showed that the ethanolic and aqueous extracts of *F. virosa* and *K. africana* contained some of these families of chemical compounds, namely alkaloids, anthocyanins, flavonoids, free quinones, tannins, and aromatic amino acids.

It is, therefore, likely that, through the synergistic effect or alone, these phytochemical groups may be at the basis of the in vitro anti-sickle cell activity of these two plants and could justify their use as anti-sickle cell plants in traditional Nigerien medicine. Thus, aqueous and ethanolic extracts of *F. virosa* and *K. africana* contain compounds that can prevent complications related to sickle cell disease, including polyphenols, flavonoids, and anthocyanins.

These compounds are known to act both on haemoglobin S polymerisation under hypoxic conditions and on the erythrocyte membrane [1]. They inhibit haemoglobin S polymerisation by engaging in a competitive reaction with this protein. In addition, their antioxidant properties enable them to prevent the peroxidation of membrane lipids, thus preventing erythrocyte lysis. Anthocyanins are effective scavengers of free radicals and are, therefore, powerful inhibitors of lipid peroxidation.

**Figure 5:**
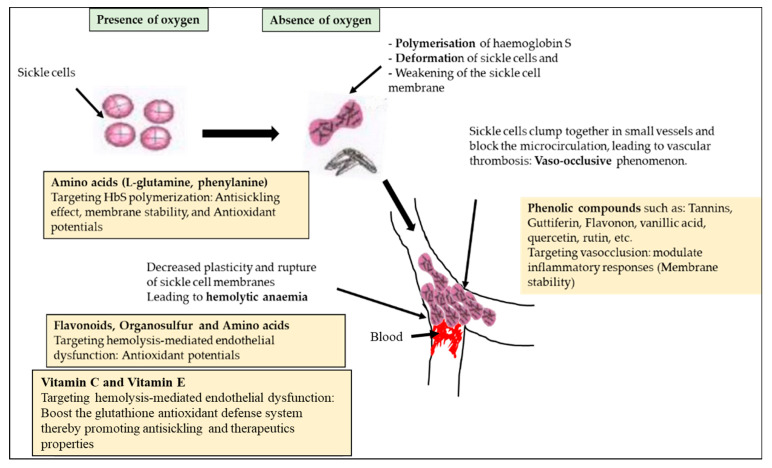
Proposed anti-sickle cell mechanisms of *F. virosa* and *K. africana* extracts.

In addition, the phytochemicals in the ethanolic and aqueous extracts of *F. virosa* and *K. africana*, owing to their antioxidant potential, are thought to target vasocclusion by modulating inflammatory responses to stabilise sickle cell membranes in order to prevent haemolysis-mediated endothelial dysfunction [34] (Figure 5).

A phytomedicine based on these compounds could protect the membranes of sickle cell patients by supplementing the enzymatic defence systems of deficient erythrocyte. They could also stabilise haemoglobin S by increasing its affinity for oxygen and promoting better circulation of water in erythrocytes, as reported by its author [35,36,37,38].

## 4. Materials and Methods

### 4.1. Chemicals and Reagents

All the organic reagents and solvents were purchased from Fisher Scientific (Strasbourg, France).

### 4.2. Biological Material

The biological material consisted of SS homozygous sickle cell haemoglobin blood collected from a patient with sickle cell disease at the National Reference Center for sickle cell disease (CNRD) at the national hospital in Niamey (Niger). This is the only centre specialized in the screening, treatment, and management of sickle cell disease. This blood sample was used to evaluate the antifalcemic activity of the crude extracts of the two plants. It was collected in EDTA tubes from a patient not recently transfused.

### 4.3. Plant Material and Preparation of Extracts

#### 4.3.1. Plant Material and the Selection Criteria for the Two Plant Species

To list the plants used in the fight against sickle cell disease in Niger, we used the results of the ethnobotanical survey carried out by [10] among traditional health practitioners in the Niamey and Tillabéri regions. This document reported the local names of plants presumed to be anti-sickle cells and the method of preparation of the recipe for the traditional medicine used to treat sickle cell disease. This enabled us to draw up a list of 23 plants. After a review of the literature, two plants whose anti-sickle cell activity had not been studied were selected. These were the aerial parts of *Flueggea virosa* (Roxb. ex Willd.) Royle and the leaves of *Kigelia africana* (lam) Benth., two medicinal plants used in the Nigerien pharmacopoeia for the treatment of sickle cell disease and other pathologies. Plant material collected from the rural commune of Liboré (in the Tillabéri region of Niger) was shade-dried, crushed, and sieved. It was identified at the Botanical Laboratory of the Biology Department of Abdou Moumouni University, Niamey. The plant powder was preserved in brown glass jars and stored in wooden cupboards.

#### 4.3.2. Preparation of the Crude Extracts of Both Plants

Plant powder (10 g) was weighed in a clean bottle using a PIONNER precision balance (to 0.001 g) (manufactured from Nänikon, Switzerland), transferred to a 250 mL Erlenmeyer flask, and 100 mL of solvent (distilled water or ethanol) was added to the powder. The mixture was macerated for 48 h at laboratory temperature (approximately 25 °C). Filtration was carried out after 48 h of maceration. The obtained macerates were filtered through cotton, and the solvents were removed using a rotary evaporator for the ethanolic extracts and a sand bath for the aqueous crude extracts to obtain the dry crude extracts. The yield (mass/mass percentage) of the crude extract was determined as follows: r = mass of dry crude extract × 100/mass of the sample powder. Before experimentation, each dry extract was stored in a brown vial and placed in a wooden cabinet to protect the photolabile compounds.

### 4.4. Phytochemical Screening

Different chemical groups (alkaloids, sterols and triterpenes, saponosides, flavonoids, free quinones, tannins, anthocyanins, reducing sugars, and aromatic amino acids) were characterized using techniques described in reference [39,40].

### 4.5. Evaluation of In Vitro Antifalcemic Activity

The Emmel technique, adapted by [41], is generally used to demonstrate the effects of plant extracts on the morphology of sickle cells.

The falciformation test for the control sample consisted of placing a drop of SS blood in a drop of physiological water (NaCl 0.9%) and sodium metabisulfite (Na_2_S_2_O_5_) between the slides. The slides were then glued to coverslips using candle wax. The mixture was depleted of oxygen, which caused red blood cells to falciform.

For the test sample, the falciformation test consists of placing a drop of SS blood in a drop of physiological water (NaCl 0.9%) and sodium metabisulfite (Na_2_S_2_O_5_) with a drop of plant extract at a given concentration (at varying concentrations: 10 mg/mL, 5 mg/mL, 2.5 mg/mL, and 0.05 mg/mL) between the slides. The slides were then glued to coverslips using candle wax. After 24 h of incubation, normal and sickle cell erythrocytes were counted via observation of the control and test slides under a light microscope with an objective (×40). The determination of sickle cells from 100 red blood cells was performed using a handheld counter [42]. Finally, the digitization of the images of sickle cell red blood cells, before and after treatment with the crude extracts of the plants, was performed using a Sony cyber-shot 10.1-megapixel digital camera manufactured from Tokyo (Japan).

### 4.6. Statistical Analyses

Data analysis was performed using the Excel 2013 spreadsheet. Analysis of variance (ANOVA) was used to test the difference between the two plants and between the two extracts in terms of normalisation rate. This analysis was performed using R software version 3.6.0 (R Development Core Team, 2019), and a *p*-value of less than 0.05 was found to be significantly different.

## 5. Conclusions

This study aimed to confirm the presumed anti-sickle cell activity of two plants from the Niger Pharmacopoeia and to determine the families of chemical compounds in their crude aqueous and ethanolic extracts. Our results confirmed the in vitro anti-sickle cell activity of *F. virosa* and *K. africana*, two plants used in traditional medicine in the Niamey and Tillabery regions, Niger, to treat SS anaemia. Anti-sickle cell activity: Starting at a dose of 0.05 mg/mL, the ethanolic extracts of the two plants normalised up to 75% of sickle cells. The greatest effects were observed in ethanolic extracts rather than aqueous extracts, with a normalisation rate for ethanolic extracts of 95% for *F. virosa* and 93% for *K. africana,* respectively, and for aqueous extracts of 90% for *F. virosa* and 85% for *K. africana*, at a concentration of 10 mg/mL. This activity could be mainly due to polyphenols, amino acids, and other organic acids, alone or with a possible synergistic effect of other families of compounds contained in the plants. Given the results, these two plants could make a significant contribution to the treatment of sickle cell disease in Niger through their use as phytomedicines.

In this study, bio-guided fractionation was performed to isolate the active ingredients and assess the toxicity of these two plants to develop a potential phytomedicine.

## Figures and Tables

**Table 1:** Yields of ethanolic and aqueous extracts of the organs of the two presumed anti-sickle cell plants.

Plants	Family	Organ Used	Extraction Mode	Plants Yield (%)
*Flueggea virosa* (Rxb. ex Wild.) Voigt	*Euphorbiaceae*	Aerial part	Aqueous	12.4
			Ethanolic	19.8
*Kigelia Africana* (Lam.) Benth.	*Bignoniaceae*	Leaves	Aqueous	11.3
			Ethanolic	5.5

**Table 2:** Phytochemical screening of two anti-sickle cell plants.

Plants	Organ	Extract	Alc.		Ster.	Fla.	Sap.	Qui.	Tan.	An.	Suc.	AA.
*Flueggea virosa* (Rxb. ex Wild.) Voigt			D	W								
	Aerial part	H_2_O	+		+	+	+	+	+	+	+	+
		EtOH	+		+	+	−	+	+	+	+	+
*Kigelia Africana* (Lam.) Benth.	Leaves	H_2_O	−		+	+	+	+	+	+	+	+
		EtOH	+		+	+	+	+	+	+	+	+

Alc. Alkaloides; D. Dragendorff; W. Wagner; Ster. Sterols and triterpenes; Fla. Flavonoids; Sap. Saponosides; Qui. Free quinones; Tan. Tannins; An. Anthocyanins; Suc. Reducing sugars; AA. Aromatic Amino Acid; +: presence of substances; −: absence of substances; H_2_O. aqueous; EtOH. ethanolic.

**Table 3:** Rate of normalisation of ethanolic and aqueous extracts from the organs of the two presumed anti-sickle cell plants.

Plants	Type of Extract	Rate of Normalization
*Flueggea virosa* (Rxb. ex Wild.) Voigt	Aqueous	90%
	Ethanolic	95%
*Kigelia africana* (Lam.) Benth.	Aqueous	85%
	Ethanolic	93%

**Table 4:** Phytochemical compounds isolated from *Flueggea virosa* [13].

Compound Name	Compound Class
(−)-securinine	Alkaloid
Virosecurinine	Alkaloid
(+)-norsecurinine	Alkaloid
(−)-virosine B	Alkaloid
(+)-virosine B	Alkaloid
Flueggine A	Alkaloid
Flueggine B	Alkaloid
Flueggether A	Alkaloid
Flueggether B	Alkaloid
Flueggether C	Alkaloid
Rutin	Flavonoid
Isoquercitrin	Flavonoid
Kaempferol	Flavonoid
Quercetin	Flavonoid
Gallocatechin	Flavonoid
Epi-gallocatechin	Flavonoid
(+)-catechin	Flavonoid
*p*-hydroxybenzoic acid	Phenolic acid
Vanillic acid	Phenolic acid
Bergenin	Polyphenol
Glucogallin	Polyphenol
Corilagin	Polyphenol

**Table 5:** Phytochemical compounds isolated from *Kigelia africana* [21].

Compound	mg/kg
Caffeic acid glucoside	38.51 ± 1.20
*p*-Coumaroyl glucose	21.40 ± 0.92
Caffeic acid	83.01 ± 1.56
*p*-Coumaric acid	27.82 ± 0.81
Ferulic acid	19.40 ± 0.67
Verbascoside	146.86 ± 3.14
Verminoside	473.88 ± 5.37
Specioside	44.39 ± 0.70
Minecoside	12.98 ± 0.43

## Data Availability

Not applicable.
